# Contribution of alcohol in accident related mortality in Belarus: a time series approach

**DOI:** 10.5249/jivr.v4i2.100

**Published:** 2012-07

**Authors:** Yury Evgeny Razvodovsky

**Affiliations:** ^a^Grodno State Medical University, Belarus.

## Abstract

**Background:**

High accidental death rates in the former Soviet republics (FSR) and its profound fluctuation over the past decades have attracted considerable interest. The research evidences emphasize binge drinking pattern as a potentially important contributor to accident mortality crisis in FSR. In line with this evidence we assume that higher level of alcohol consumption in conjunction with binge drinking pattern results in close aggregate-level association between alcohol psychoses and accidental death rates in the former Soviet Slavic republic Belarus.

**Methods:**

Trends in alcohol psychoses rate (as a proxy for alcohol consumption) from 1979 to 2007 were analyzed employing a distributed lag analysis in order to asses bivariate relationship between the two time series.

**Results:**

According to the Bureau of Forensic Medicine autopsy reports the number of deaths due to accidents and injuries increased by 52.5% (from 62.3 to 95.0 per 100.000 of residents), and fatal alcohol poisoning rate increased by 108.6% (from 12.8 to 26.7 per 100.000 of residents) in Belarus between 1979 and 2007. Alcohol in blood was found in 50.1% victims of deaths from accidents and injuries for the whole period, with the minimum figure 40% in 1986 and maximum 58.2% in 2005. The outcome of distributed lags analysis indicated statistically significant association between the number of alcohol psychoses cases and the number BAC-positive deaths from accidents at zero lag.

**Conclusion:**

The outcome of this study supports previous findings suggesting that alcohol and deaths from accidents are closely connected in a culture with prevailing intoxication-oriented drinking pattern, and add to growing body of evidence that a substantial proportion of accidental deaths in Belarus are due to effects of binge drinking.

## Introduction

It is widely recognized that acute alcohol intoxication is associated with an increased risk of different types of accidents.^1,2^Excessive alcohol consumption is a major cause of considerable number of fatal traffic accidents throughout the world.^3,4,5^In many countries alcohol also plays a significant role in accidental falls, accidents caused by fire, accidental drowning.^6,7^

There is evidence of a strong association between alcohol and accidental deaths at individual level. In a Czech study, using materials of forensic autopsies, alcohol in the blood has been detected in 34.7% victims of fatal traffic accidents.^8^ Alcohol has been found to be related to 44, 49, and 37% of accidental deaths among Finish males in the age groups 15-34, 35-54 and 55-59 years.^9^A review of studies based on Norwegian data suggests that about 35% of male fatal accidents were alcohol-related.^10^ In the US, alcohol may have caused 29% of all unintentional fatal injuries and more than 40% fatal motor vehicle crashes.^11^ It was estimated that 45% of all accidental deaths among males 15-69 years were alcohol-related in northern Europe; the alcohol attribute fraction for southern and central Europe was 40% and 35% respectively.^12^

A substantial literature exists on the association between alcohol and non-fatal injury.^13^The findings from emergency rooms studies usually reported a greater prevalence of higher rates of alcohol use disorders among injured compared to non-injured patients. In particular, approximately 45% of trauma patients have reported alcohol misuse based on screening with the CAGE and SMAST questionnaires.^14^ In another study three fourths of acutely intoxicated traumatic patients had evidence of chronic alcoholism as indicated by a positive SMAST, and approximately 35% had biochemical evidence of chronic alcohol abuse.^15^ The outcomes of the study conducted at Oulu University Hospital (Finland) suggest that 77% patients injured by falls on the ground and 94% injured by motor vehicle crash were BAC-positive.^16^ The study of alcohol and injury in the emergency service of a public hospital in Warsaw found that among injured 35% males and 12% females reported seven or more drinks during the 6 hour prior to injury.^17^ In a prospective cohort study conducted in the US was shown that 47% of trauma patients had a positive BAC and 35.8% were intoxicated (BAC more than 100 mg/dl).^15^Overall, a review of international emergency rooms studies found positive BAC estimates among injured patients to range from 6% to 32%, while self-reported consumption within 6 hour prior to the event ranged from 8% to 39%.^13^

Several case-control studies in emergency department have shown a dose-response relationship for the risk of injury. McLeon et al. (1999) reported results from a case-control study investigating the relationship between alcohol use and injury in Australia.^18^ Their results suggest that the risk of sustaining an injury increased by three-fold after consuming more than 60 g of alcohol and five times after consuming more than 90 g of alcohol. In a Mexico City study, it was demonstrated a general trend of an increase in the estimated relative risk with an increase in consumption above one drink during the 6 hour period prior injury.^19^ In the large sample of patients with non-fatal injuries attending emergency departments worldwide were found that the risk of injury increased with consumption of a single drink and there was a 10-fold increase for patients who had consumed six or more drinks during the previous 6 hours.^20^In fact, this is the first case-crossover study to show that having only a one drink is associated with a non-fatal injury.

There are several studies indicating that binge drinking is associated with high risk of trauma.^21^Savola et al. (2005) reported that binge drinking is a major risk factor for head trauma among trauma patients and that the relative risk for head injury markedly increases with increasing blood alcohol level.^16^ In recent study from Finland was shown an excess of head traumas during weekends and this excess was associated with heave episodic drinking.^22^Similar weekly variations of head trauma have been reported in other countries where heavy episodic drinking is also the prevailing drinking pattern.^23^

The strong support for a causal role of alcohol in accidental mortality comes from aggregate-level studies. Both longitudinal and cross-sectional aggregate-level studies have reported elsewhere a significant temporal co-variation between per capita alcohol consumption and accidental mortality rates.^24^ An analysis of time-series data for Canada covering the period 1950-1998 revealed a statistically significant association between per capita alcohol consumption and overall fatal accidents rates.^25^ In a cross-sectional time-series analysis based on data from 50 American states, population drinking remained a predictor of traffic fatalities even after controlling for potential confounders.^26^Nevertheless, the cross-country comparisons demonstrate heterogeneity with respect to strength of association between population drinking and accidental mortality.^24^ In countries where high level of intoxication is an integral part of the drinking culture, the etiological significance of alcohol seems to be larger. A time series analysis, based on the data for the period from 1950-95 covering 14 European Union countries suggests that an increase in population drinking had large impact on accident mortality in northern Europe than in mid-Europe and southern Europe.^27^These findings provided support for the hypothesis, that the effect of alcohol on accidents mortality rate is stronger in the northern European spirits countries characterized by a low per capita consumption with the bulk of consumption concentrated on a few occasions (binge drinking pattern), or "dry" drinking cultures, than in the southern European wine countries with a high average consumption which is more evenly distributed throughout the week, or "wet" drinking cultures. Similarly, the results of recent study suggest that changes in per capita consumption have a significant impact on injury mortality in 6 eastern European countries, but the strength of the association tends to be stronger in countries where intoxication-oriented drinking pattern prevails.^28^

There is common believe that high level of alcohol consumption in conjunction with binge drinking pattern is a major determinant of accident mortality crisis in the former Soviet republics (FSR).^29^ The findings suggest that population drinking and accidental deaths rates are positively related phenomena in Russia.^30^ There is also suggestive evidence that in the FSR accident mortality rate is more responsive to per capita changes in distilled spirits consumption than the total level of alcohol consumption. The results from recent time series analysis based on Russian data from 1980 to 2005 suggest that 1 liter increase in overall alcohol sale would result in a 4.6% increase in the accident mortality rate, while a 1 liter increase in vodka sales is expected to increase the number of deaths from accidents and injuries by 11%.^31^ Similar results have been reported in the time-series analysis based on Belarusian data from the period 1970–2005.^32^ It was highlighted that 1 liter increase in alcohol sale is associated with a 6.2% increase in the number of deaths from accidents and injuries; a 1 liter increase in vodka sales would result in a 10.7% increase the accidental mortality rate.

Additional development of the idea that alcohol can have detrimental effect on violent mortality in FSR came from individual level studies. A recent study of 22658 forensic autopsies, performed in the Siberian city of Barnaul during 1990–2004 has shown that among autopsied aged 35-69 years who were reported to have died from external causes 76% of men and 65% of women were BAC-positive and 25% of men and 24% of women had blood concentration of alcohol 4 g/l or more.^33^

The level of alcohol consumption and the accidental mortality rate in the former Soviet Slavic republic Belarus are both among the highest in the world.^32^ As a predominantly spirits drinking country, Belarus is characterized by infrequent, but heavy (binge) drinking leading to high rates of acute alcohol-related problems. Although alcohol seems to be an important contributor to the burden of violent mortality in Belarus, little systematic research has been undertaken on its impact on mortality from accidents in this country. The aim of the present study was to address this particular deficit by using Bureau of Forensic Medicine autopsy data on the deaths from accidents and injuries between 1979 and 2007.

## Methods

**Data**

The data on deaths due to accidents (fatal occupational injuries, traffic accidents, accidental falls, accidents caused by fire, accidental drowning) used in the article were based on autopsy reports from Bureau of Forensic Medicine. In Belarus, virtually all (99.1%) violent deaths are subjected to forensic autopsies, which include blood alcohol concentration (BAC) inspection. BAC in samples collected by forensic pathologists during the medical autopsies was assessed by gas chromatography and reported per million (0/00). BAC over 0.5 0/00 was termed "inebriation" and denoted as "BAC-positive". National statistical agencies cause-of- death classification has been subjected to several changes over the last decades. Between 1970 and 1988, Ministry of Statistics used the coding scheme based on ICD-8, and in 1989, ICD-9 was introduced. In 2002, a new coding system, based on ICD-10, came into practice. The Belarusian coding system is claimed to be compatible with ICD-9 and ICD-10.^31^

In present study we used the alcohol psychoses incidence rate as a proxy for the aggregate level of alcohol consumption. The alcohol psychoses incidence as an indicator of harmful drinking may capture more effectively the magnitude of alcohol-related problems among the population than official sales statistics and expert’s estimation of the total level of alcohol consumption.^32^ This is particularly true if we keep in mind that reliable estimation of total alcohol consumption at the population level in the former Soviet republics is highly questionable.^30^We specified the number of persons admitted to the hospital for the first time for the treatment as incidence of alcohol psychoses: (ICD–10: F 10). Alcohol psychosis is a secondary psychosis with predominant hallucinations occurring in many alcohol-related conditions, including acute intoxication, withdrawal, after a major decrease in alcohol consumption. In ICD-10,^34^the diagnosis of alcohol psychosis can be made if the following criteria are fulfilled:

1. Onset of psychotic symptoms must occur during or within 2 weeks of alcohol abuse.

2. The psychotic symptoms must persist for more than 48 hours.

3. Duration of the disorder must not exceed 6 month. The data on alcohol psychoses incidence rate (per 100.000 of the population) are taken from the Ministry of Statistics of Belarus annual reports for the years from 1979 to 2007.

**Statistical analysis**

The statistical analysis was performed using the package "STATISTICA". It is generally agreed that bivariate correla-tions between two raw time-series are spurious due to common sources of trends and autocorrelation.^24^ Therefore in order to reduce the risk of obtaining a spurious relation between two variables that have common trends, the trends should be removed by means of a differencing procedure: Ϫxt = xt - xt-1.35 This means analyzing annual changes rather than raw data. The process of removing systematic variation within time series prior to the examination of potential causal relationships is referred to as "prewhitening". The residuals of a statistically adequate time series are distributed as a white noise process. A further step entails the inspection of the cross-correlation function in order to estimate the association between the two prewhitened time series. We used the distributed lags analysis to estimate the relationship between the time series deaths due to accidents and injuries and alcohol psychoses rate (as a proxy for alcohol consumption) in this paper.

## Results

According to Bureau of Forensic Medicine autopsy reports the number of deaths due to accidents increased by 52.5% (from 62.3 to 95.0 per 100.000 of residents) in Belarus between 1979 and 2007. The number of BAC-positive deaths due to accidents increased by 71.2% (from 29.5 to 50.5 per 100.000 of residents) and number of BAC-negative deaths increased by 35.7% (from 32.8 to 44.5 per 100.000 of residents). Alcohol in blood was found in 50.1% victims of deaths from accidents for the whole period, with the minimum figure 40% in 1986 and maximum 58.2% in 2005. 

**Figure 1: Trends in the number of BAC-positive and BAC-negative cases of deaths from accidents in Belarus between 1979 and 2007:**
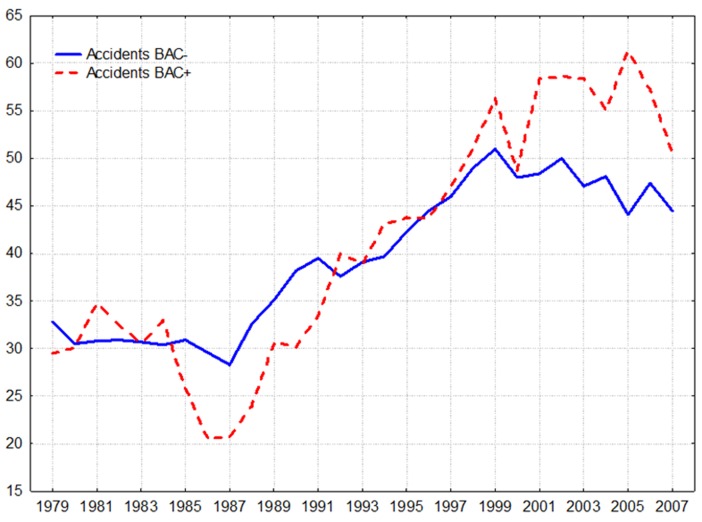


A comparative analysis show that trend in BAC-positive deaths due to accidents tends to fluctuate across time series to a much greater extent that the BAC-negative deaths rates (Figure 1). Alcohol-related mortality from accidents was more affected by the restriction of alcohol availability during the anti-alcohol campaign: between 1984 and 1987 the number of BAC-positive deaths due to accidents drop by 37.9% (from 33 to 20.5 per 100.000 of residents), while number of BAC-negative deaths decreased by 6.9% (from 30.4 to 28.3 per 100.000 of residents). Further, the upward trend in BAC-positive accident mortality in 1990s was greater than trend in BAC-negative deaths from accidents and injuries: from 1987 to 1999 the number of BAC-positive deaths increased by 174.6% (from 20.5 to 56.3 per 100.000 of residents), while the number of BAC-negative deaths increased by 80.2% (from 28.3 to 51 per 100.000 of residents).

The trends of BAC-positive deaths due to accidents and alcohol psychoses rates are displayed in Figure 2. As can be seen, there is quite a strong association between the two time series. The two time trends fluctuated over the period: dropped sharply in 1984-1987, began to increase in 1988, dramatically jumped from 1991 to 1998. In 1999/2000 there was a slight decrease in the rates and from 2000 it again began to rise until 2004, than started to decrease in the last years.

**Figure 2: Trends in the number of BAC-positive cases of deaths from accidents (R) and the number of alcohol psychoses cases (L) in Belarus between 1979 and 2007.**
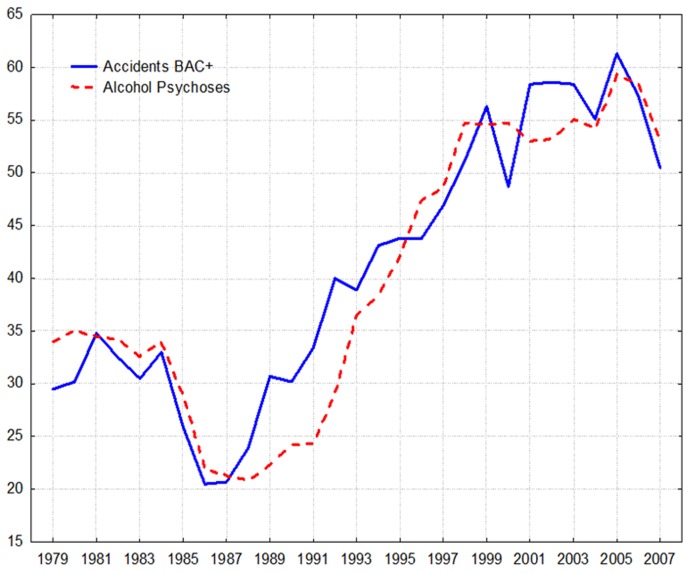


As can be seen from Figure 1there is linear trend in the time series. This trend was removed by means of first-order differencing procedure. After pre-whitening the cross-correlations between alcohol psychoses and accident mortality time series were inspected. The outcome indicated statistically significant cross-correlation between alcohol psychoses rate and the number BAC-positive deaths from accidents and injuries (r=0.48; SE 0.189) at zero lag (Table 1).At the same time, there is no relation between the alcohol psychoses and BAC-negative accidental deaths. The outcome of the distributed lags analysis is presented in Table 2. It can be seen, that the estimated effect of alcohol psychoses rate (as a proxy for alcohol consumption) on the number BAC-positive deaths due to accidents and injuries is statistically significant at zero lag.

**Table:** Table 1: **The results of cross-correlation analysis between prewhitened time series of alcohol psychoses and accident mortality rates**

Lag	Alcohol psychoses/BAC+ Accidents		Alcohol psychoses/BAC- Accidents	
	r	SE	r	SE
-3	-0.011	0.200	0.044	0.200
-2	-0.231	0.196	-0.161	0.196
-1	0.312	0.193	0.353	0.193
0	0.483	0.189	-0.033	0.189
1	0.377	0.193	0.043	0.193
2	-0.023	0.196	0.243	0.196
3	0.123	0.200	0.323	0.200

**Table:** Table 2:**The results of distributed lags analysis between time series of alcohol psychoses and accident mortality rates**

Lag	Regres Coeff	Standard Error	t	p
0	0.351	0.136	2.623	0.015
1	0.243	0.143	1.874	0.325
2	-0.325	0.219	-0.039	0.306
3	-0.068	0.242	-0.249	0.831

## Discussion

The findings from the present study suggest that alcohol drinking and BAC-positive accident mortality rates are positively related phenomena in Belarus: Gorbachev’s anti-alcohol campaign 1985–1988 was associated with a rapid reduction in the level of alcohol consumption and the number of BAC-positive fatal accidents and injuries, while increasing alcohol consumption in the transitional period has been linked to increase in accident mortality rates.

There is a suggestion that the decrease in the number of deaths from accidents and injuries in the former Soviet republics in the mid-1980s could have been related to the political and social liberalization during the period known as "perestroika", which gave rise to social optimism and new hope.^36^Nevertheless, the results of present study suggest that the number of BAC-positive accidental deaths shrank by 37.9%, while the number of BAC-negative deaths due to accidents and injuries did not change substantially during Gorbachev’s perestroika. This fact in conjunction with the coincident trends between the alcohol psychoses rate and the number of BAC-positive deaths from accidents in the mid-1980s indicate that a restriction of alcohol availability can be considered as an effective measure of accident mortality prevention.

Several scholars have argue that psychosocial distress resulting from the "shock therapy" economic reform and sudden collapse of the Soviet paternalist system was the main determinant of the violent mortality crisis in the former Soviet republics in the 1990s.^37,38^They tried to capture the transition-related stress by using combined measurement of these hypothetical stressors. For example, Cornia and Poniccia (2000) illustrated that the psychosocial stress, measured by shift in the Gini coefficient, explained the greatest part of the variance in violent mortality for the European regions of Russia.^39^ In recent study was highlighted that in post-communist countries of Eastern Europe and the former Soviet Union rapid mass privatization as an economic transition strategy was associated with a short-term increase in mortality rates in working-aged men .^40^

To address hypothetical role of psychosocial distress in accidental mortality crisis it is necessary to focus on the social and economic changes that occurred in Belarus in the 1990s. The collapse of communism and the initial moves to establish a market economy resulted in the newly independent country experiencing a severe economic and social crisis. Between 1991 and 1995 real gross product fell by over 30%, inflation had reached more than 2300%, and unemployment rose substantially.^41^Against this background, the level of poverty rose sharply while increasing social dislocation was manifested in falling birth and marriage rates and growing number of divorces.^32^So, psychosocial distress may be an important underlying factor of accident mortality crisis the 1990s. However, the fact that the number of BAC-positive deaths due to accidents dramatically jumped in the 1990s strongly supports an alcohol related hypothesis and suggests that rather that playing major causal role, psychosocial distress may represent a confounding factor.

It seems plausible that the psychosocial distress resulting from the reforms were the main causes of increased demand for alcohol at this time. This demand was met by factors that increased supply. Following the repeal of state alcohol monopoly in 1992, Belarusian"s alcohol market became fragmented, including many private producers and importers operating without a license or registration. The country was practically flooded by a wave of homemade, counterfeit, and imported alcohol of low quality. In the second half of the 1990s, the overall level of alcohol consumption grew to 14-14.5 liters per capita, the highest rate in the country’s history.^32^ The negative outcomes of increase of alcohol consumption during this period included a sharp rise in accident mortality.

Before concluding, it is necessary to consider the potential limitations of this study. This principally applies to the quality of the data used. An earlier study of violent mortality data from the Soviet period concluded that they were not deliberately falsified in Belarus.^42^while a recent study has argued that comparisons can be made across time in the country using Soviet and post-Soviet data.^43^Further, there exists no assessment of the accuracy of hospital admission data. However, it’s fairly close resemblance with the temporal pattern of alcohol poisoning mortality seems reassuring, as an earlier study confirmed the reliability of the mortality statistics for the Soviet and post-Soviet periods.^42^In addition, the author’s discussions with psychiatrist did not reveal any differences in the way in which alcohol psychoses are being diagnosed and recorded in contemporary Belarus.

In conclusion, this study suggests a close link between alcohol psychoses (as a proxy for alcohol consumption) and mortality due to accidents at the aggregate level. The outcome of this study also supports previous findings suggesting that alcohol and deaths from accidents and closely connected in culture with prevailing intoxication-oriented drinking pattern and add to growing body of evidence that a substantial proportion of accidental deaths in Belarus are due to acute effect of binge drinking. The results of present study, as well as findings from other settings indicate that a restrictive alcohol policy can be considered as an effective measure of accidental deaths prevention in countries where rates of both alcohol consumption and accident mortality are high.
